# Supplementary material for: Modeling Dragons: Using linked mechanistic physiological and microclimate models to explore environmental, physiological, and morphological constraints on the early evolution of dinosaurs
Source: PLoS One. 2020 May 29;15(5):e0223872. doi: 10.1371/journal.pone.0223872 (PMC7259893; doi:10.1371/journal.pone.0223872)
Supplement: S1 Appendix — (PDF) [file pone.0223872.s001.pdf]

Niche Mapper Variables

The number of variables (691; representing all data for 1 day each month for 1 year) in the combined microclimate and physiological models seems large. However, the microclimate model can be broken into 13 discrete categories. For instance, the microclimate model may boast 198 variables, but the vast majority represent the range of a given category, such as minimum and maximum values for each modeled day (24 states). In this way 5 categories (relative humidity, air temperature, cloud cover, wind, and percent shade) account for 60% of microclimate variables. In other words, there are 24 variables for relative humidity (a high and low for each of the 12 modeled days of the year), etc.

The biophysical model can be broken into 12 categories for body shape (99 variables), 10 categories for heat generation and transient properties (179 variables), 4 categories for dietary/digestive/water properties (131 variables), and 4 categories for behavior (89 variables).

Microclimate model variables

Model Parameters	Quantity	Comments:
Number of days to model	12	A start, stop, and day number are chosen (from 1-365)
Substrate conditions	8	Roughness, conductivity,

		density, heat, depth
Surface object profiles (bushes, rocks, etc.)	4	Experimental wind profiles Set to zero if none measured.
Geographic Information	10	Hemisphere, latitude, longitude, slope, azimuth, elevation,
Maximum/Minimum % shade	24	Potentia shadel range for model to choose from
Timing of maximum minimum	8	(e.g., air/wind max~solar noon; relative humidity/cloud~sunrise
Maximum/Minimum relative humidity	24	Aridity/humidity (min/max per modeled day)
Maximum/Minimum cloud cover	24	Insolation (min/max per modeled day)
Maximum/Minimum wind speed	24	(min/max per modeled day)
Maximum/Minimum air temperature	24	(min/max per modeled day)
Snow or free water presence/absence	12	
Substrate reflectivity (albedo)	12	
% of surface acting like free water	12	

Allometry (modeled organism dimension properties)

Model Parameters	Quantity	Comments:
Animal group/locomotion	2	Clade, and biped vs. quad
Head geometry	9	Length:width:height; fur depth (dorsal and ventral); density and head geometry
Neck geometry	9	
Torso geometry	9	
Front legs	9	

Back legs	9	
Tail/additional appendage geometry	9	(this is same as above but can be used to model a trunk as well)
Total length/scaling adjustments	3	
Subcutaneous fat distribution/mass change	7	Distribution and region(s) of subcutaneous fat storage
Postures	7	Start/End postures for shade, sleep, and inactive (modeled body parts in contact with ground or as single lump)
Flesh conductivity (head-tail) (min)	13	Also accounts for torso overhang (shading legs)
Core temperature relation with appendages	8	average core temperature that is somewhere between ground and torso core temperature

Physiological and behavioral properties

Model Parameters	Quantity	Comments:
General coding and output parameters	10	
Heat storage; specific heat	6	Transient vs stead state; specific heat for flesh
Mass, subcutaneous fat, density, appendages	11	Whole body parameters, number of appendages; substrate interaction, fur/feather compression when lying down
Metabolic rate; activity multiplier; thermoregulation triggering	4	*how the model uses heat generated from activity
Forage rate; muscle efficiency; production heat	4	Contribution of activity to heat balance model

Insulation properties: head, neck, torso, tail dorsal/ventral	33	Diameter, length, depth, density, reflectivity,
Hair/feather length/depth dorsal/ventral	48	*allows for seasonal changes
Temperature and water loss from skin and metabolism	15	Core temp: min, max, target; skin wet/sweat, pilo/ptiloerect; skin/fur thermal conductivity; depth within fur/feather for radiant exchange
Core temperature (target) (hibernation)	24	Tc per modeled day
Time dependant change in mass/body fat	24	For each modeled day
Lungs and gut	5	O2 extraction min/max; gut passage time; fecal and urea,
Digestive efficiency	12	For each modeled day
Times basal for activity energy & food for it (1-7)	24	This can be used to simulate phenology of food available and reproduction timing.
Food: %protein,carbs, fat, dry matter	48	
Diurnal, nocturnal, crepuscular, hibernate (fraction of day hibernating)	60	For each modeled day
Active/inactive on land or water	24	For each modeled day
Thermoregulation behavior	13	Use nest; burrow shade; climb, wind protection; night shade; dive; wade; thermoregulation order
Flight, burrow, nest properties, shelter	34	Specific to nest, flight,and burrowing behaviors including shape and thermal properties of structures and (burrow gas properties)
