# Supplementary material for: Modeling Dragons: Using linked mechanistic physiological and microclimate models to explore environmental, physiological, and morphological constraints on the early evolution of dinosaurs
Source: PLoS One. 2020 May 29;15(5):e0223872. doi: 10.1371/journal.pone.0223872 (PMC7259893; doi:10.1371/journal.pone.0223872)
Supplement: S2 Appendix — (PDF) [file pone.0223872.s002.pdf]

Model test using *Varanus komodoensis* on Komodo Island

Although Niche Mapper has been tested for reptiles ranging in size from small iguanids [1-3] to land iguanas in the Galapagos [4] and small and large birds [5-7] and mammals [8-10] we include here an additional test using the Komodo dragon, *Varanus komodoensis*, on Komodo Island. We obtained data for monthly climates for this location using Adagio in niche-mapper.com to point-and-click on Komodo Island to extract climate data from New and others [11]. Komodo morphology/proportions were obtained from web photos of wild komodos from Komodo Island and implemented in the alomvars.dat input file for Niche Mapper. Key physiological properties, e.g. maximum and preferred core temperatures, were obtained from Harlow and others [12]. We set the model to allow 24 hour activity, diurnal only, and diurnal + crepuscular to see if there were any noticeable differences in energy requirements.

We assumed a high-level squamate metabolism and a skin reflectivity of 15% based on black reptile skin (Galapagos marine iguana) reflectivities measured by WPP. Since the allometry subroutines in Niche Mapper will automatically scale the allometry isometrically, given the mass and a reference dimension, e.g. snout-vent length or shoulder height, we were able to run annual simulations for a 6.7 kg and 65 kg animal assuming a density of 1030 kg/m^3 for both animals, and compute the body core temperatures, activity patterns and food requirements for the month of November when observations recorded in the literature were made that could be used for comparison to the simulations [13,14]. **Figure 1 in S2 Appendix** illustrates the computed body temperatures that would occur with a squamate metabolism for the large, 65 kg, and small, 6.7 kg Komodo dragon. The measured shade air temperatures reported for this time are also plotted.

Computed activity hours, patterns of annual activity in sun/shade and energy and food requirements for the 65 kg dragon exhibit a consistent pattern of energy and food requirements while activity hours varied under three activity conditions: diurnal only, diurnal-crepuscular, and 24 hour potential activity (**Fig. 2 in S2 Appendix**). For comparison, a simulation of the 65kg dragon was conducted within the Triassic hot microclimate model to illustrate the impact on activity, shade use, and food requirements under our deep-time conditions (**Fig. 3 in S2 Appendix**). We also ran a simulation for an allometrically modified 6.7 kg dragon, since the young have apparent longer legs and narrower torso than adults to determine allometry effects (Fig. 2 in **S2 Appendix**).

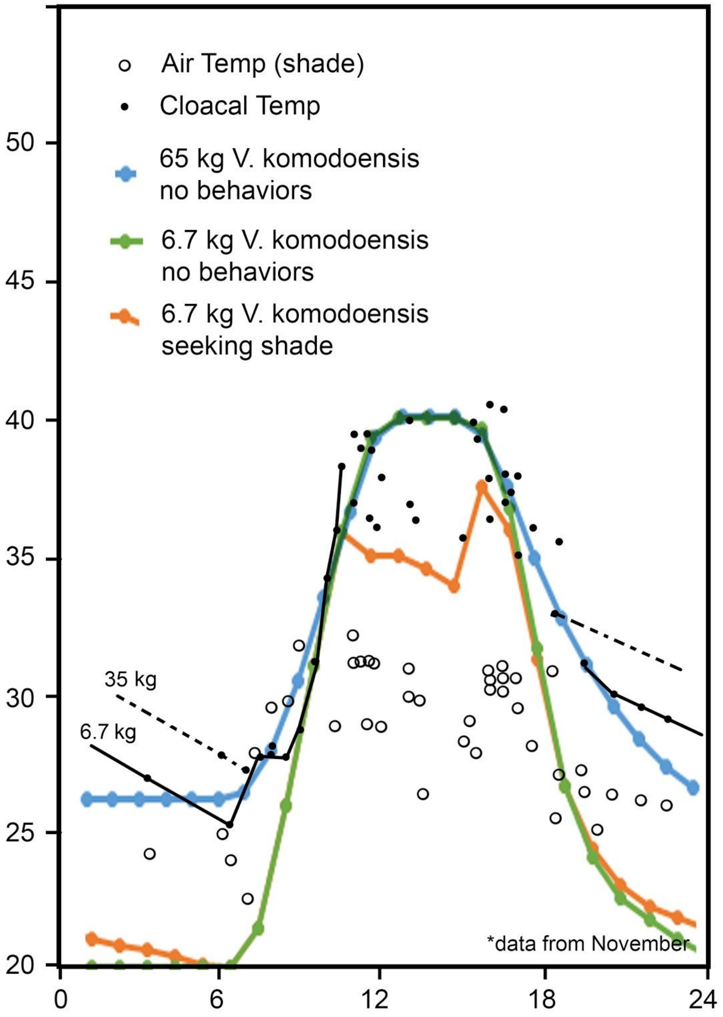

Figure 1. These data represent field cloacal temperatures measured from 28 individuals (closed circles) and heating and cooling curves from a 6.7 (solid black line) and 35 kg (dashed line) *V. komodoensi*s on Komodo Island including shaded air temperatures (open circles); modified after McNab and Auffenberg [13]. The 65kg *V. komodoensis* modeled in NicheMapper (blue line) demonstrates a similar hourly temperature pattern to the empirical data. The modeled 6.7kg *V. komodoensis* (green line) predicts a cooler night time temperature than available empirical data. Empirical data for the 6.7 kg animal is not recorded between the hours of 20:00 and 06:00 and and may be due to fossorial behaviour. The modeled 6.7kg *V. komodoensis* with thermoregulatory behaviors enabled (orange line) predicts a slightly warmer nighttime core temperature (seeking night shade) and cooler mid-day temperatures (seeking shade).

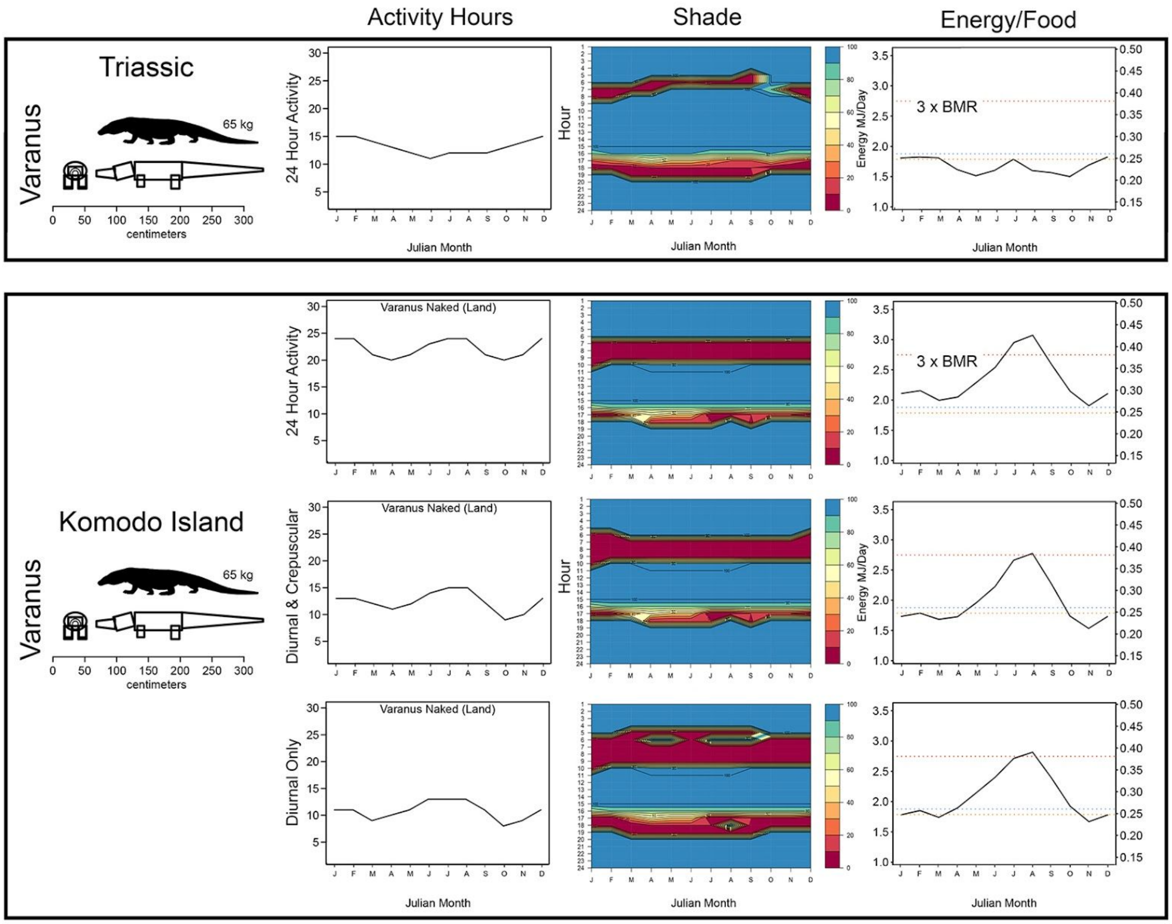

Figure 2. The 65 kg *V. komodoensis* was modeled in 4 different scenarios, 1 under hot Triassic conditions with 24 hours potential activity and 3 under Komodo Island conditions with a 24 hour, diurnal, and diurnal + crepuscular potential activity. In the 'Energy/Food' column, the increase in energy requirements mid-year on Komodo Island represents the cooler temperatures of the southern hemisphere winter. The modeled shade ranges from 0-100% (red-blue, respectively), with night shade representing the animal retreating from open air conditions to reduce net radiant heat loss to the night sky and cold ground surface, and day shade representing retreat to overhanging vegetation due to excessive core body temperatures. The modeled values for shade seeking behavior on Komodo Island reflect the observed bimodal activity peaks in the morning and evening [14]. *V. komodoensis* modeled in the Triassic hot monsoon microclimate exhibits a similar pattern to that seen on Komodo Island, though the activity hours over a 24 hour cycle and the energy requirements are shifted toward more nocturnality, likely due to higher T_{air}.

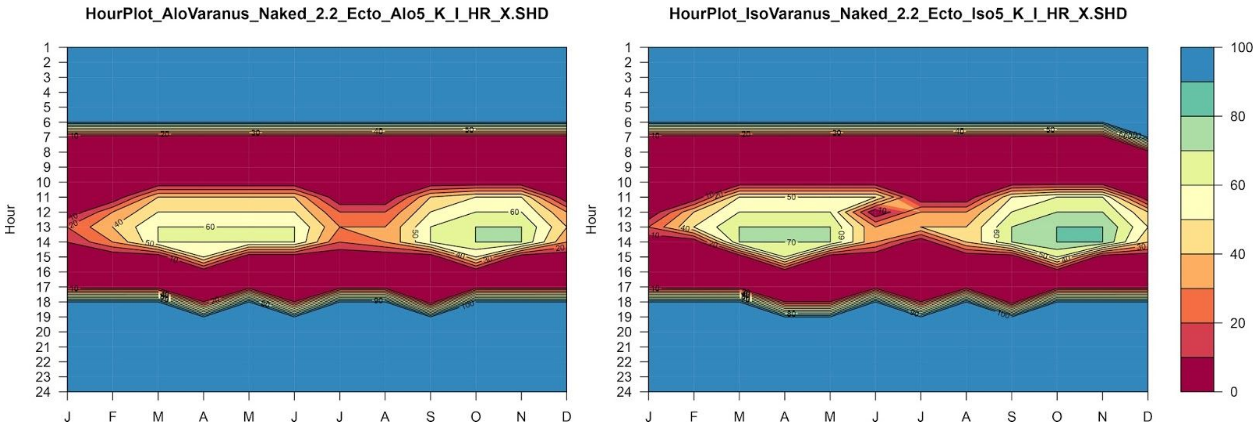

Figure 3. Allometric (left) vs isometric (right) scaling of juvenile Komodo dragon at 6.7 kg from an adult Komodo dragon of 65 kg. Young dragons have smaller bodies and longer legs relative to adult Komodo dragons. These graphs illustrate subtle (but minimal) changes in the amount of shade needed during the middle of the day in the hot times of the year on Komodo Island south of the equator.

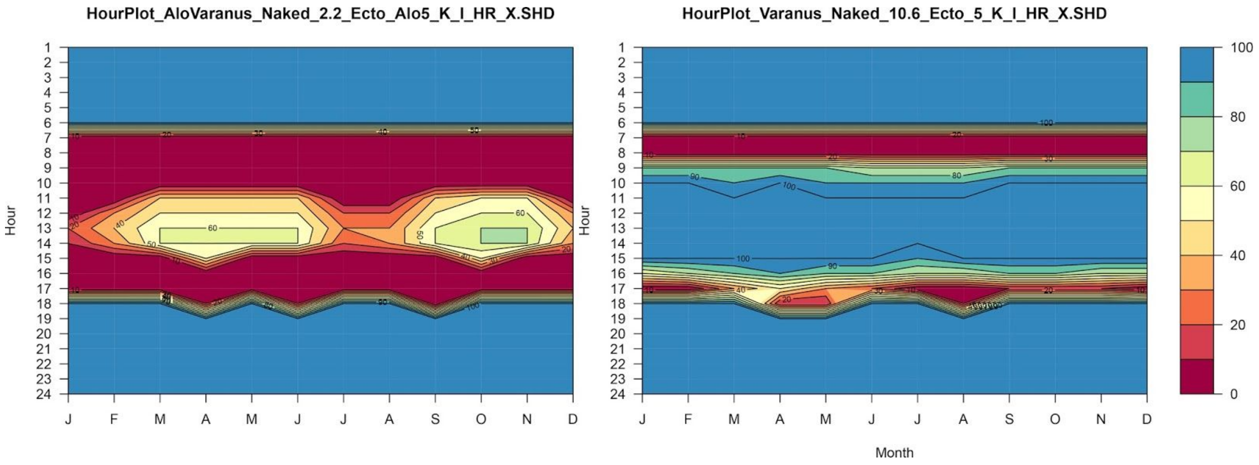

Figure 4. Computed 6.7 kg juvenile Komodo dragon activity pattern (left) vs. an adult 65 kg Komodo dragon activity pattern (right). These graphs illustrate the behavioral separation of activity patterns of small vs. large Komodo dragons during the day and throughout the year on Komodo Island south of the equator.

References

1. Porter WP, Mitchell JW, Beckman WA, DeWitt CB. Behavioral implications of mechanistic ecology: Thermal and behavioral modeling of desert ectotherms and their microenvironment. Oecologia. 1973; 13: 1-54.
2. Grant BW. Porter WP. Modeling global macroclimatic constraints on ectotherm energy budgets. Amer Zoologist. 1992; 32(2): 154-178.
3. Jones SM, Ballinger RE, Porter WP. Physiological and Environmental Sources of Variation in Reproduction: Prairie Lizards in a Food Rich Environment. Oikos. 1987; 48(3): 325-335.
4. Christian K, Tracy CR, Porter WP. Seasonal shifts in body temperature and use of microhabitats by Galapagos land iguanas (Conolophus pallidus). Ecology.1983; 64(3): 463-468.
5. Porter W, Vakharia N, Klousie W, Duffy D. Po'ouli landscape bioinformatics models predict energetics, behavior, diets, and distribution on Maui. Integr Comp Biol. 2006; 46(6): 1143-1158.
6. Fort J, Porter WP, Grémillet D. Thermodynamic modelling predicts energetic bottleneck for seabirds wintering in the northwest Atlantic. J Exp Biol. 2009: 212(15): 2483-2490.
7. Fitzpatrick MJ, Mathewson PD, Porter WP. Validation of a mechanistic model for non-Invasive study of ecological energetics in an endangered wading bird with counter-current heat exchange in its legs. PLoS One. 2015; 10(8): e0136677.
8. Porter WP, Munger JC, Stewart WE, Budaraju S, Jaeger J. Endotherm energetics: from a scalable individual-based model to ecological applications. Aust J Zool. 1994; 42: 125-162.
9. Conley KE, Porter WP. Heat loss from deer mice (*Peromyscus*): evaluation of seasonal limits to thermoregulation. J Exp Biol, 1986; 126(1): 249-269.
10. Porter WP, Kearney M. Size, shape, and the thermal niche of endotherms. Proc Nat Acad Sci. 2009; 106(Supplement 2): 19666-19672.
11. New M. et al., A high-resolution data set of surface climate over global land areas. Climate research. 2002; 21(1): 1-25.
12. Harlow HJ, Purwandana D, Jessop TS, Phillips JA. Body temperature and thermoregulation of Komodo dragons in the field. J Therm Biol. 2010; 35(7): 338-347.
13. McNab BK, Auffenberg W. The effect of large body size on the temperature regulation of the Komodo dragon, Varanus komodoensis. Comp Biochem Phys A. 1976; 55(4A): 345-350.
14. Auffenberg W. The behavioral ecology of the Komodo monitor. 1st ed. Gainesville:University Presses of Florida;1981.
