# Supplementary material for: Modeling Dragons: Using linked mechanistic physiological and microclimate models to explore environmental, physiological, and morphological constraints on the early evolution of dinosaurs
Source: PLoS One. 2020 May 29;15(5):e0223872. doi: 10.1371/journal.pone.0223872 (PMC7259893; doi:10.1371/journal.pone.0223872)
Supplement: S3 Appendix — (PDF) [file pone.0223872.s003.pdf]

Parameterizing biophysical model dimensions for fossil vertebrates

Niche Mapper calculates its hourly heat balance equations utilizing a simplified geometric approximation of research taxa (Fig. 1), allowing for large numbers of simulations to run on relatively modest hardware. The results of this approach have been verified by comparison to high resolution models subject to large-scale fluid dynamics simulations in Ansys Fluent [1,2]. In extant taxa such as *Varanus komodoensis*, parameterization of physical dimensions relies on direct or reported measurements. Extinct taxa require additional steps to ensure sufficient dimensional accuracy.

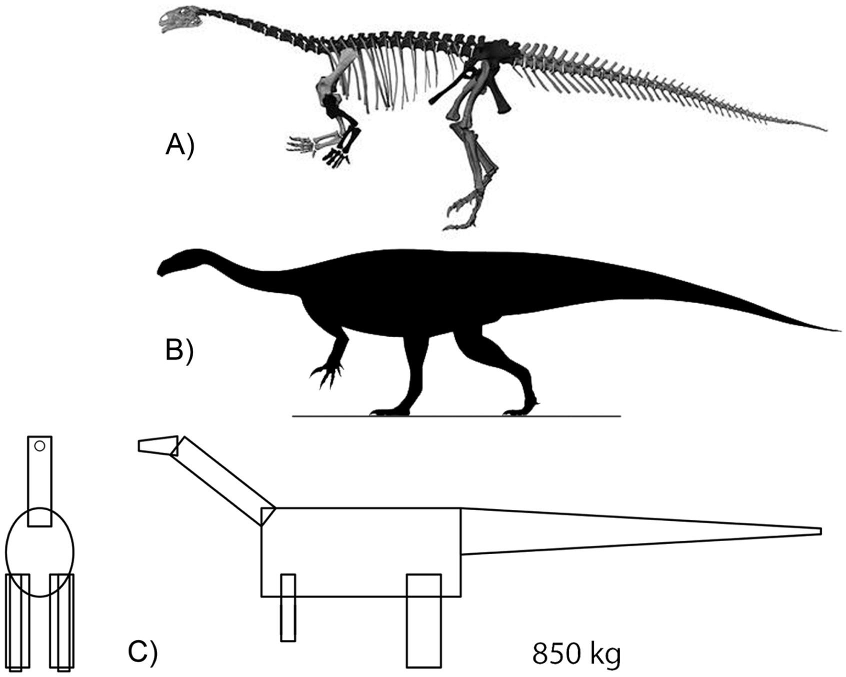

Figure 1. Modeled volume for *Plateosaurus*.

Simplified overview of steps followed to create simplified geometric models for Niche Mapper biophysical input. Linear dimensions are taken from fossil data such as (A) this surface scan of GPIT125 [3]. That data is used for mass estimates, e.g. (B) lateral view silhouette used in GDI mass estimate, and the data is input to create a geometrically simplified (C) Niche Mapper model.

Dimensional inputs required by Niche Mapper include linear measurements and estimate of specific gravity for the head, neck, limbs, torso, and tail. An independent mass estimate is useful for comparison to the mass generated by Niche Mapper from the input dimensions and specific gravity of each body segment, as a check on potential errors in data input.

Dimensional parameters required by Niche Mapper are based on proportions of living animals. For *Coelophysis* and *Plateosaurus* estimates of life dimensions started with dimensionally accurate skeletal reconstructions (Fig. 2). Linear dimensions of individual skeletal elements were obtained from direct measurement of specimens and published data, as detailed in Wang, et al. [4]. The largest impact to dimensional proportions are competing interpretations of pectoral girdle placement [5].

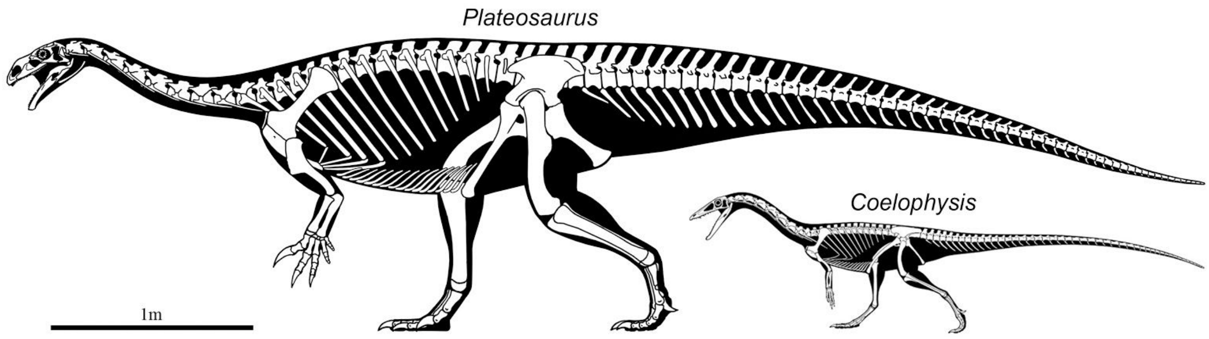

Figure 2. Skeletal reconstructions of *Plateosaurus* and *Coelophysis*.

Mass estimates for dinosaurs have a long history in paleobiology [e.g. 6], and have been attempted via disparate methods including limb bone allometry [7,8], volumetric measurements of scale models [9,10,11], graphic double integration [12], computational application of minimum convex hulls [13] and various other dimensional analyses based on CT skeletal data [3,14,15,16]. The approach used in Niche Mapper is also a volumetric computational one, where total mass and mass distribution are calculated within Niche Mapper by assigning model volumes and densities for each body segment. Niche Mapper mass results were checked against Graphic Double

Integration of rigorous skeletal reconstructions for *Coelophysis* and *Plateosaurus* [Fig. 2; 12,17] as well as previously published mass estimates [11,15]. Diet composition was inferred from dental morphology. Skin transpiration and breathing efficiency were estimated from extant analogs.

References

1	Dudley PN, Bonazza R, Porter WP. Consider a Non-Spherical Elephant: Computational Fluid Dynamics Simulations of Heat Transfer Coefficients and Drag Verified Using Wind Tunnel Experiments. J Exp Biol. 2013; 319(6): 319-327.
2	Dudley PN, Bonazza R, Porter WP. Climate change impacts on nesting and interesting leatherback sea turtles using 3D animated computational fluid dynamics and finite volume heat transfer. Ecol Model. 2016; 320: 231-240.
3	Mallison H. The digital Plateosaurus II: an assessment of the range of motion of the limbs and vertebral column and of previous reconstructions using a digital skeletal mount. Acta Palaeontologica Polonica. 2010 Sep;55(3):433-58.
4	Wang X, Pittman M, Zheng X, Kaye TG, Falk AR, Hartman SA, Xu X. Basal paravian functional anatomy illuminated by high-detail body outline. Nature communications. 2017 Mar 1;8:14576.
5	Hartman S. Investigating the impact of competing interpretations of pectoral girdle placement and appendicular function on sauropod head height. Journal Of Vertebrate Paleontology 2012 Sep 2 (Vol. 32, pp. 106-106).
6	Gregory WK. The Weight of the Brontosaurus. Science. 1905; 22(566): 572.
7	Seebacher F. A new method to calculate allometric length-mass relationships of dinosaurs. J Vertebr Paleontol. 2001; 21(1): 51-60.
8	Peczkis J. Implications of body-mass estimates for dinosaurs. J Vertebr Paleonol. 1995; 14(4): 520-533.
9	Alexander RM. Dynamics of dinosaurs and other extinct giants. Columbia University Press. 1989.
10	Colbert EH. The weights of dinosaurs. Amer. Mus. Novit. 1962; 2076: 1-16.
11	Paul GS. Dinosaur models: The good, the bad, and using them to estimate the mass of dinosaurs. In: Wolberg DL, Stumpand E, Rosenberg, GD, editors. Dinofest International. Proceedings of a symposium held at Arizona State University.1997;129-142.
12	Murray PF, Vickers-Rich P. Magnificent mihirungs: the colossal flightless birds of the Australian dreamtime. Indiana University Press; 2004.

13	Sellers, W.I., Hepworth-Bell, J., Falkingham, P.L., Bates, K.T., Brassey, C.A., Egerton, V.M. and Manning, P.L., 2012. Minimum convex hull mass estimations of complete mounted skeletons. Biology Letters, 8(5), pp.842-845.
14	Gunga HC, Suthau T, Bellmann A, Friedrich A, Schwanebeck T, Stoinski T, Trippe T, Kirsch K, Hellwich O. Body mass estimations for Plateosaurus engelhardti using laser scanning and 3D reconstruction methods. 2007. Naturwissenschaften; 94(8): 623-630.
15	Mallison H. The Digital Plateosaurus I: Body mass, mass distribution and posture assessed using CAD and CAE on a digitally mounted complete skeleton. Palaeon Electron. 2010; 13(2): 8A 26p.
16	Hutchinson, J.R., Bates, K.T., Molnar, J., Allen, V. and Makovicky, P.J., 2011. A computational analysis of limb and body dimensions in Tyrannosaurus rex with implications for locomotion, ontogeny, and growth. PLoS One, 6(10), p.e26037.
17	Jerison HJ. Gross brain indices and the meaning of brain size. Evolution of the Brain and Intelligence. New York, Academic Press. 1973:55-81.
