# Supplementary material for: Modeling Dragons: Using linked mechanistic physiological and microclimate models to explore environmental, physiological, and morphological constraints on the early evolution of dinosaurs
Source: PLoS One. 2020 May 29;15(5):e0223872. doi: 10.1371/journal.pone.0223872 (PMC7259893; doi:10.1371/journal.pone.0223872)
Supplement: S4 Appendix — (PDF) [file pone.0223872.s004.pdf]

Sensitivity Analyses

The strength our modeled results, in part, relies on understanding how sensitive the model is to ranges of values for variables that are not directly measurable in deep-time. As such, we endeavor to demonstrate that many of the variables (main effects and interactions) have relatively small effects on overall metabolic needs of the modeled organisms. However, we realize these effects can be cumulative and are more significant at the boundaries of a modeled organisms' temperature tolerance where small changes can be the difference between survival or death. Variables that have a more significant impact, such as temperature, CTR, RMR, and insulation are presented with a range of inputs for each experiment, so that results can be compared and interpreted appropriately. The following sensitivity analyses were conducted to quantify the advantage or disadvantage our chosen values would impart on the model.

Skin and insulation reflectivity:

Reflectivity of the skin and epidermal insulatory structures for our organisms are modeled at 15% (0.15) which approximates a dark coloration. Reflectivity of black and white pelts of dairy cows are documented to be around 0.16 and 0.48, respectively (pers. observation, WP). To test the effect of color selection *Coelophysis* and *Plateosaurus* were modeled with various reflectivities (0.1, 0.15, 0.2, 0.25, and 0.6; see Fig. 5). There was little effect across this range with a fully insulated *Coelophysis* which is demonstrated to be near its target metabolic energy levels (~1400 MJ/y) under the

cold microclimate. Reducing insulatory covering to top-only and non-insulated individuals produced an energetic benefit of lower reflectivity (e.g., darker color) in the cold microclimate - the more cold stressed, the larger the effect. The benefit of darker color is still swamped by the overall cold-stress the modeled organisms experience in the cold microclimate (e.g., >5 x RMR). *Plateosarus* did not show a significant change in annual metabolic need with varying reflectivity. This suggests that thermoregulation behaviors are effective at moderating the effects of reflectivity regardless of color when an organism is near its target ME, and benefits of coloration only become realized well past the boundaries of tolerable temperatures.

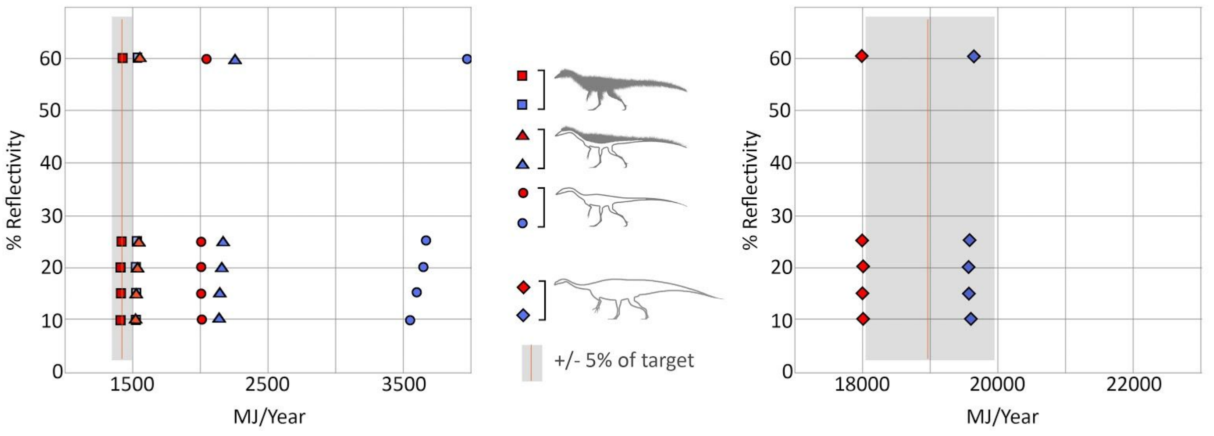

Figure 1. Skin/insulation color sensitivity analysis.

The grey shaded area represents +/- 5% of the annual target ME. The further to the right of the target zone the greater the cold stress. Filled shapes: blue = cold microclimate, red = hot microclimate. The filled blue circles (uninsulated *Coelophysis*) exhibit the greatest advantage in decreased reflectivity due to extremely cold stress. When the modeled organism (e.g. fully insulated *Coelophysis*) is within its target, the effect of color is greatly minimized. *Plateosaurus* exhibits little to no benefit with changing reflectivity.

Muscle efficiency:

Most mammals, regardless of size, have a muscle efficiency of 0.25-0.30, and the vast majority of muscle efficiency for vertebrate clades ranges between 0.2 and 0.4 [1]; an efficiency factor of 0.2 in Niche Mapper (i.e., 20%) means that 80% of activity-generated energy is lost as heat instead of powering the activity. We chose to model our dinosaur's muscle efficiency at 20% for all experimental runs. To test the impact of increased active muscle efficiency (e.g., decreasing muscle heat lost during activity) we ran experiments with 20, 30, 40, and 50% efficiency (Fig. 6). When the modeled organism is near its target metabolic energy the disparity from low to high efficiency is around 2.5% in the hot microclimate and 5-7% in the cold microclimate. The greater the cold stress the modeled organism experienced the greater the disparity between 20 and 50% muscle efficiency.

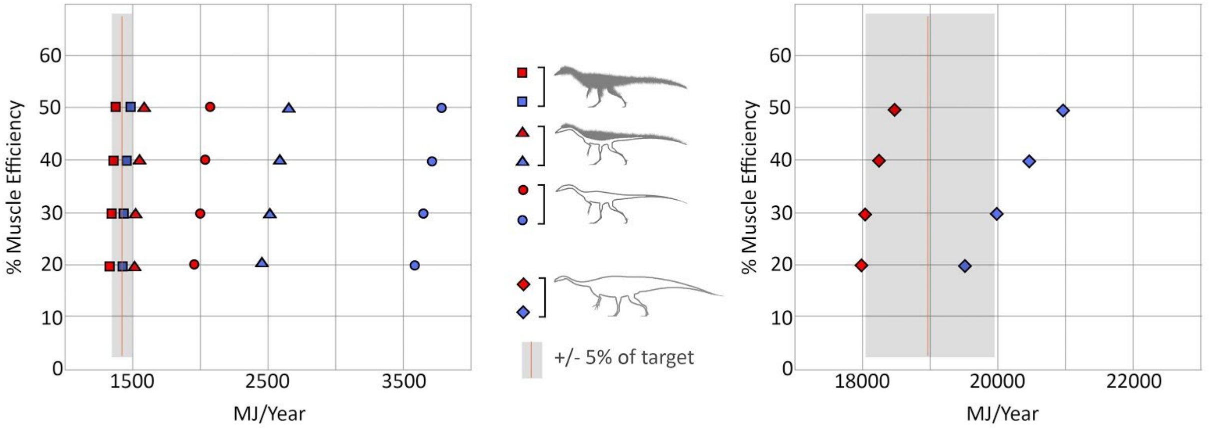

Figure 2. Muscle efficiency sensitivity analysis.

The grey shaded area represents +/- 5% of the annual target ME. Filled shapes: blue = cold microclimate, red = hot microclimate. Fully insulated *Coelophysis* meets its target regardless of assigned muscle efficiency. As muscle efficiency is increased *Plateosaurus* is less heat stressed in the hot microclimate, but more cold stressed in the cold microclimate.

Respiratory efficiency:

Oxygen extraction efficiency (EO_2) is used to calculate respiratory heat and water loss. When the organism is overheating one behavioral parameter in Niche Mapper allows the model to mimic panting by decreasing EO_2 to ‘force’ the organism to breathe more rapidly leading to greater heat (and water) loss. Phylogenetic bracketing would suggest that early saurischian dinosaurs had a avian-like unidirectional airflow supported by numerous air sacs throughout the respiratory tract [2-4]. The range of EO_2 values for avian lungs is much greater than other non-volant vertebrates (20-60%; [5]) We chose to use a 20% maximum and 15% minimum EO_2, which is in line with values reported for ratites and many falconiformes (21-26% respectively; [5]). To test the effect of EO_2 values we varied the max/min across four ranges 10/5, 20/15, 30/25, and 31/15; the last value range was to see if the max/min disparity had a noticeable effect. Varying the oxygen efficiency or max/min disparity had negligible effect on the annual energy budget (Fig. 7).

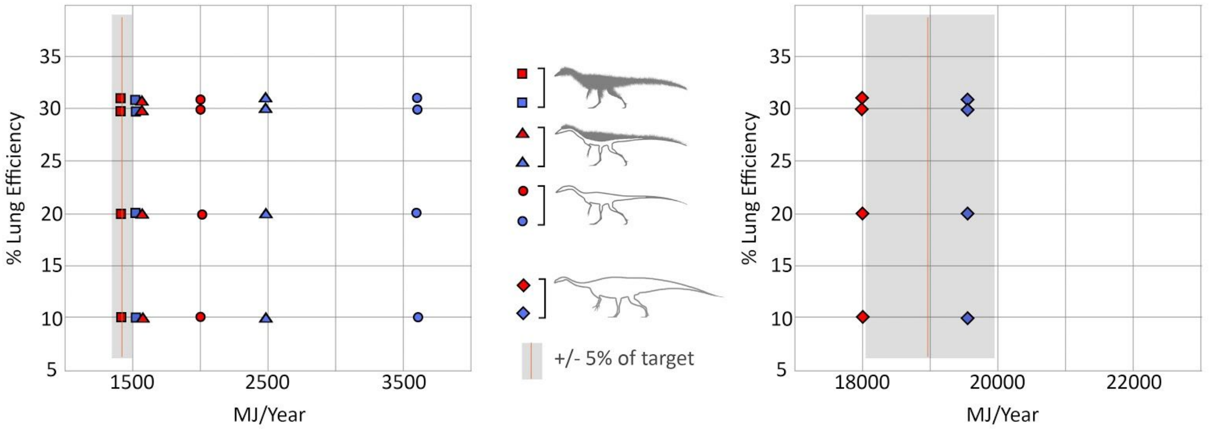

Figure 3. Respiratory efficiency sensitivity analysis.

The grey shaded area represents +/- 5% of the annual target ME. Filled shapes: blue = cold microclimate, red = hot microclimate. Varying the lung efficiency had minimal effect on annual ME.

Digestive efficiency:

We modeled *Coelophysis* with a crocodile like digestive efficiency at 85% which is only slightly higher than the average digestive efficiencies of many birds of prey (75-82%; [6]). We varied digestive efficiency between 70 and 85% for our carnivorous taxon to determine the effect on total wet-food intake on an annual basis. *Plateosaurus*, modeled as an herbivore, was assigned a 60% digestive efficiency which is below the 70% efficiency seen in ratites [7], but above 50-55% seen in some herbivorous lizards as well as passerines on a low-quality diet [8,9]. Others have argued for sauropod digestive efficiencies to be as low as 33% on a low-quality diet [10]. We tested a digestive efficiency range of 30-70% for our herbivorous diet.

As is the case with all dietary parameters in Niche Mapper varying digestive efficiency had no impact on the calculated metabolic energy (e.g., calculations for metabolic energy are independent of dietary calculations). The results (Fig. 8) are based on our assigned nutrient values (percent fat, carbohydrates, protein and dry mass; see Table 3) of the food source for the high browsing herbivorous and carnivorous diet. Changing the digestive efficiency of *Plateosarus* from 70 to 50% (a reasonable range with phylogenetic bracketing) resulted in a 70% increase total wet-food mass; the modeled *Plateosaurus* would need to ingest 3500 to 5000 kg (70-50% digestive efficiency respectively) on an annual basis. At the lower extreme, a 30% digestive efficiency would require ~8000 kg wet-food per year; this is ~22 kg of

wet-food per day, which is on par with similarly sized extant browsing mammals such as the black rhinoceros [11,12].

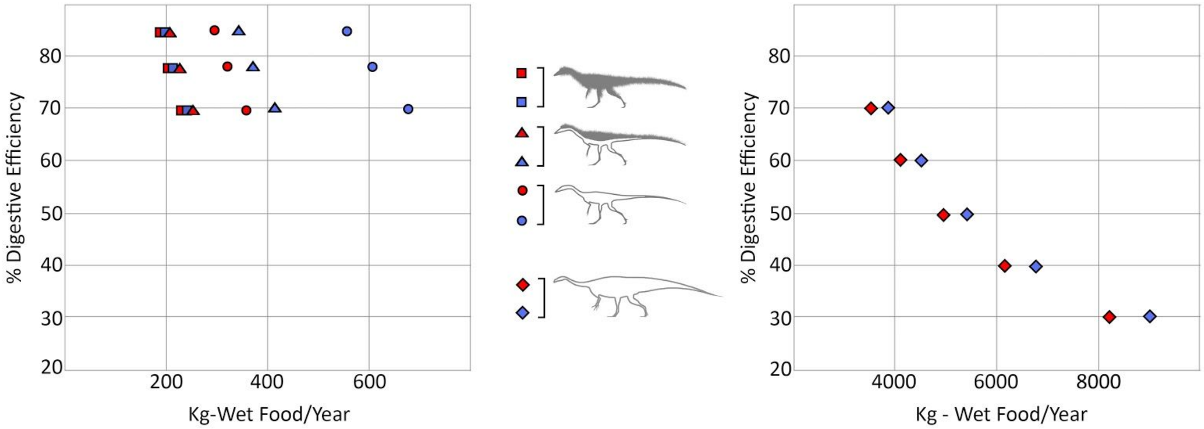

Figure 4. Digestive efficiency sensitivity analysis.

Filled shapes: blue = cold microclimate, red = hot microclimate. Varying the digestive efficiency had no effect on annual ME, however it greatly impacted the annual quantity of wet food consumption.

Effects of latitude:

The cold microclimate we use for our experiments is intended to be a lower boundary representative of more temperate latitudes during the Late Triassic; these values are consistent with GCM models [13] for 45°N. However, to test the effect of insolation at higher latitudes we modeled our organisms at 45°N paleolatitude, in addition to the 12°N paleolatitude of the Chinle Formation we use to derive most of our microclimate model data. The primary effect of increasing latitude to 45°N appears to have been a result of increased daylight hours midyear and decreased daylight hours during the winter months. This is most apparent in the increased hours/day that core temperature was maintained, midyear, and decreased during the winter months relative

to those observed at 12°N (Fig. 12). The model is more sensitive to microclimate temperatures than variance in insolation due to increased latitude. The remainder of the study uses insolation values from 12°N paleolatitude, and treats the cold microclimate as a surrogate for temperate latitudes. As more paleoclimate proxy data becomes available for higher latitudes this can be more rigorously tested.

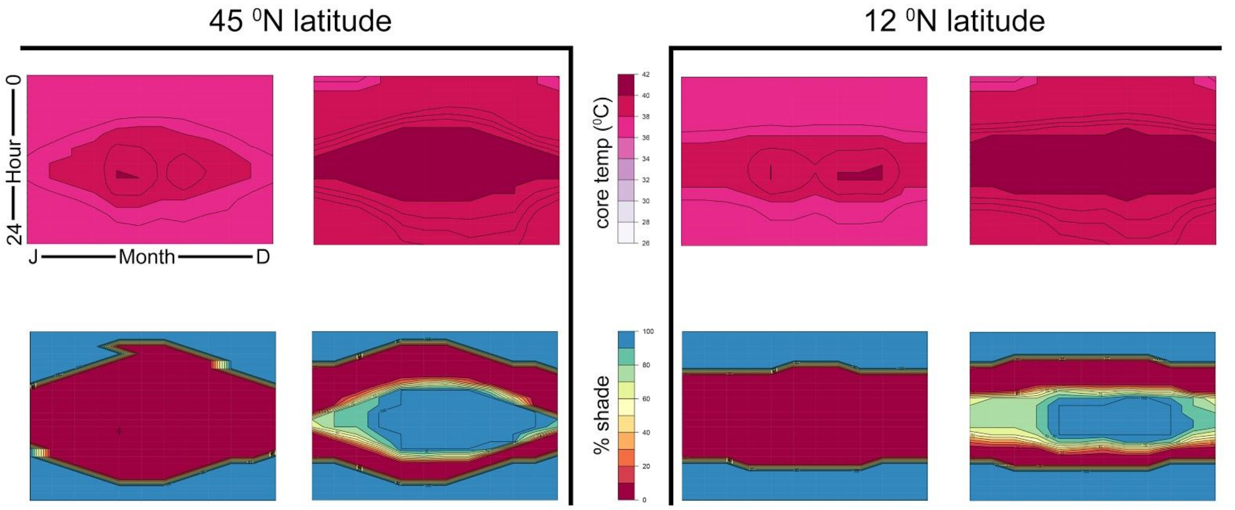

Figure 5. Effect of latitude (*Plateosaurus*) sensitivity analysis.
Heat maps of core temperature (top) and shade utilization (bottom) demonstrating the increase in daylight hours midyear and decrease in daylight hours during the winter months at high (45°N) latitude relative to low (12°N) latitude.

Yates Analyses of climate parameters:

To test the main and interactive effects of four primary climate parameters (temperature, humidity, wind speed, and cloud cover) a 2^4 factorial design and Yates' algorithm [14] was analysed (Fig. 13). The results show that temperature has the largest effect on our modeled organisms' annual energy budget. The fully insulated *Coelophysis* was modeled with and without the behavioral ability to ptiloerect. With

ptiloerect enabled, *Coelophysis* was less affected by wind speed - likely due to an increased boundary layer provided by ‘fluffing’ up the insulation layer; temperature had an order of magnitude more effect than any other climate variable for the fully insulated *Coelophysis*. Wind speeds had the second greatest effect, while humidity and cloud cover were both negligible. The uninsulated *Coelophysis* and *Plateosaurus* had similar responses to the four climate parameters; temperature was still the dominant effect, but only by a factor of 2. Because temperature and insulation have a large effect on the results, we include all insulatory states, as well as temperature ranges for all of the following analyses.

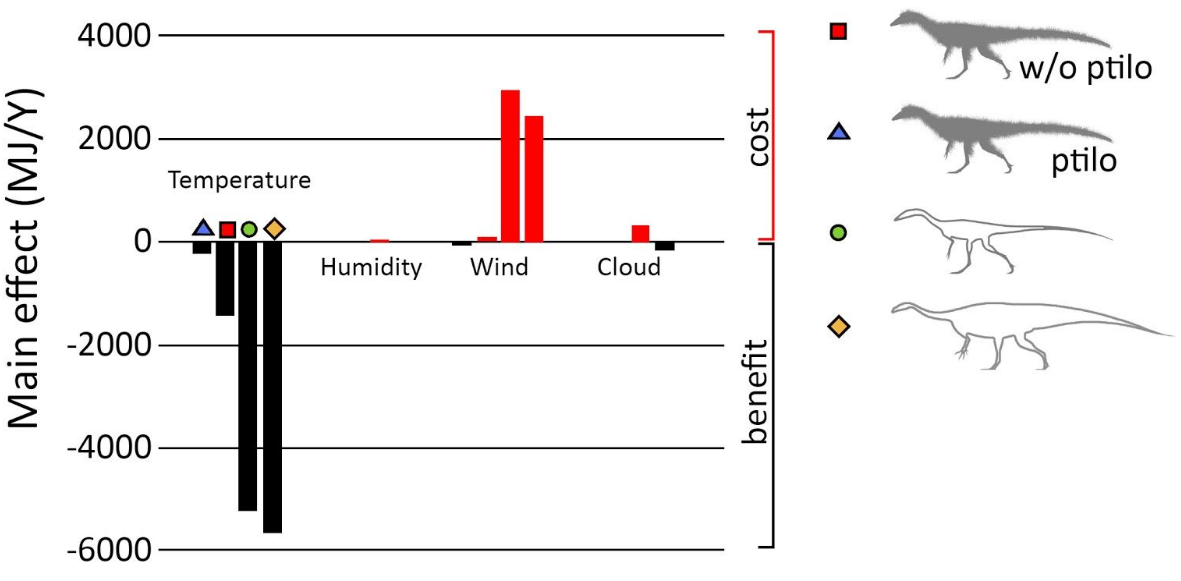

Figure 6. Yates analysis of climate parameters.

Climate parameters temperature, humidity, wind, and cloud cover demonstrate the strong effect temperature has on annual metabolic energy. Wind was the second most significant effect, while humidity and cloud cover were both negligible. Note: both uninsulated models (*Coelophysis* and *Plateosaurus*) were strongly affected by temperature and wind; the insulated models were not as greatly impacted.

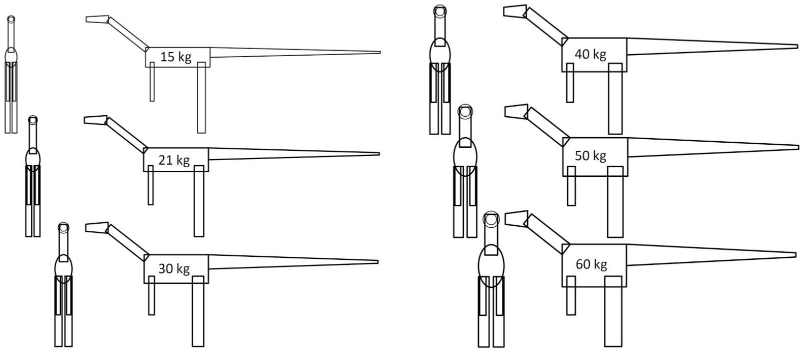

Figure 7. ***Coelophysis*** **mass estimate sensitivity analysis.**

Six models were generated for a 15, 21, 30, 40, 50, and 60 kilogram *Coelophysis*.

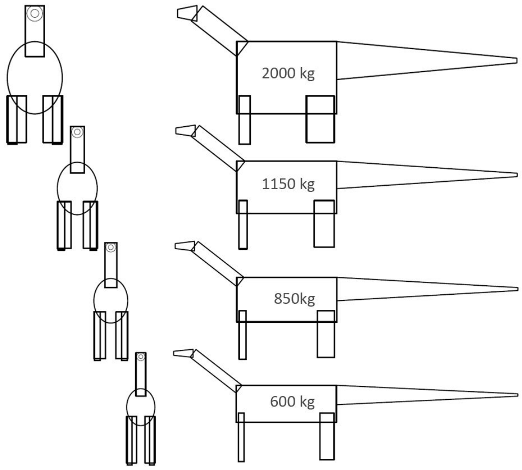

Figure 8. ***Plateosaurus*** **mass estimate sensitivity analysis.**

Outlines of the Niche Mapper models represent the 600, 850, 1150, and 2000 kilogram *Plateosaurus*.

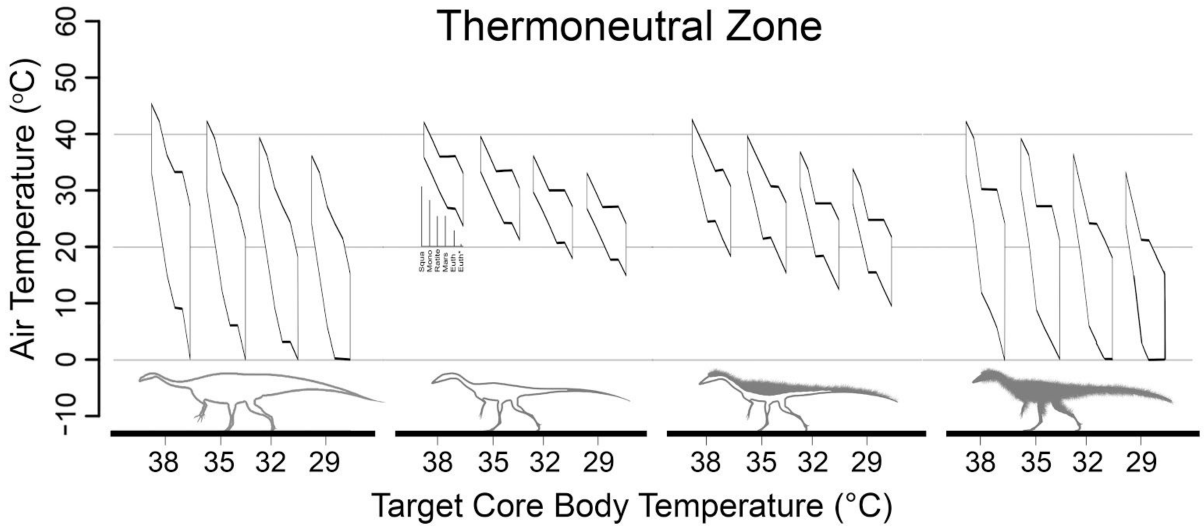

Figure 9. Effect of variable *target* core temperatures (narrow CTR).

Active thermoneutral zones of 6 metabolic rates, left to right for each of the 16 polygons, (Squa = squamate [15], Mono = monotreme [16], Mars = marsupial [16], Ratite = ratite [17], Euth = eutherian [16], Euth*=eutherian [15] at four different target core body temperatures with a narrow core temperature range (+/- 2 °C). The thermoneutral zones for the lowest target core temperature for *Plateosaurus* and the lowest two for the fully insulated *Coelophysis* extend below zero °C, but our analyses stopped at 0°C. References [15,16,17] are in the main text.

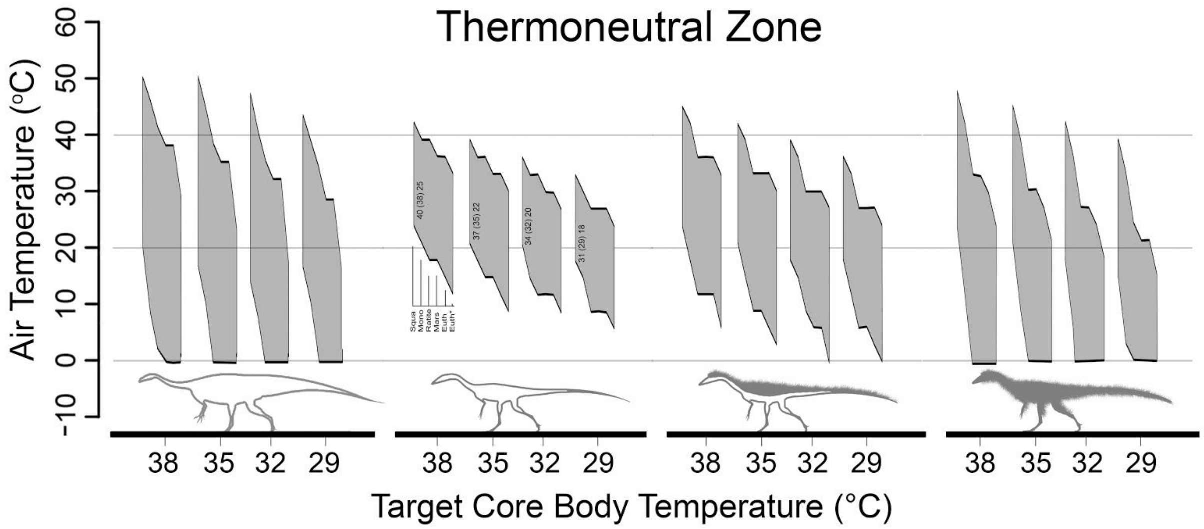

Figure 10. Effect of variable *target* core temperatures (broad CTR).

Active thermoneutral zones of 6 metabolic rates, left to right for each of the 16 polygons, (Squa = squamate [15], Mono = monotreme [16], Mars = marsupial [16] Ratite = ratite [17], Euth = eutherian [16], Euth*=eutherian [15] at four different target core body temperatures with a broad core temperature range (+2/-13 degrees C). The thermoneutral zones for *Plateosaurus* and the fully insulated *Coelophysis* extend below zero °C, but our analyses stopped at 0°C. References [15,16,17] are in the main text.

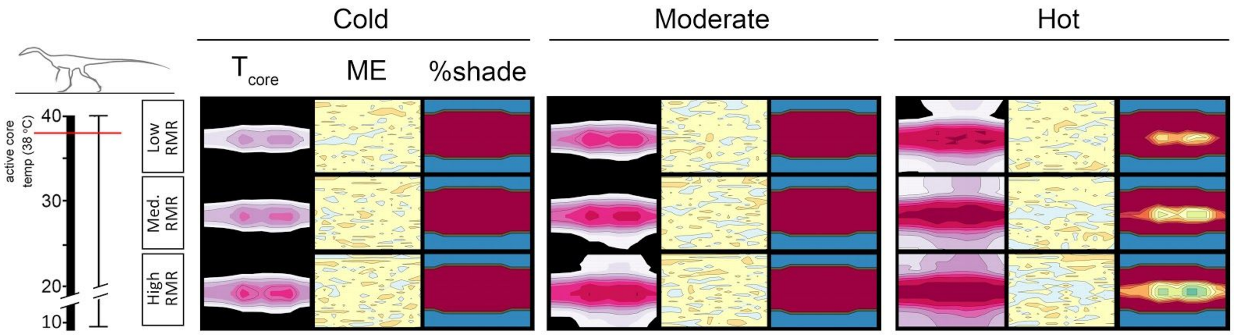

Figure 11. Heatmap of extended CTR for *Coelophysis* (uninsulated).
Extending the lower boundary of the broad CTR allowed *Coelophysis* to maintain its target ME (+/- 5% of 2xRMR). However, T_{core} does not exceed 30°C for more than 5 months of the year in the cold microclimate. Black color in the T_{core}= temperatures below 26°C.

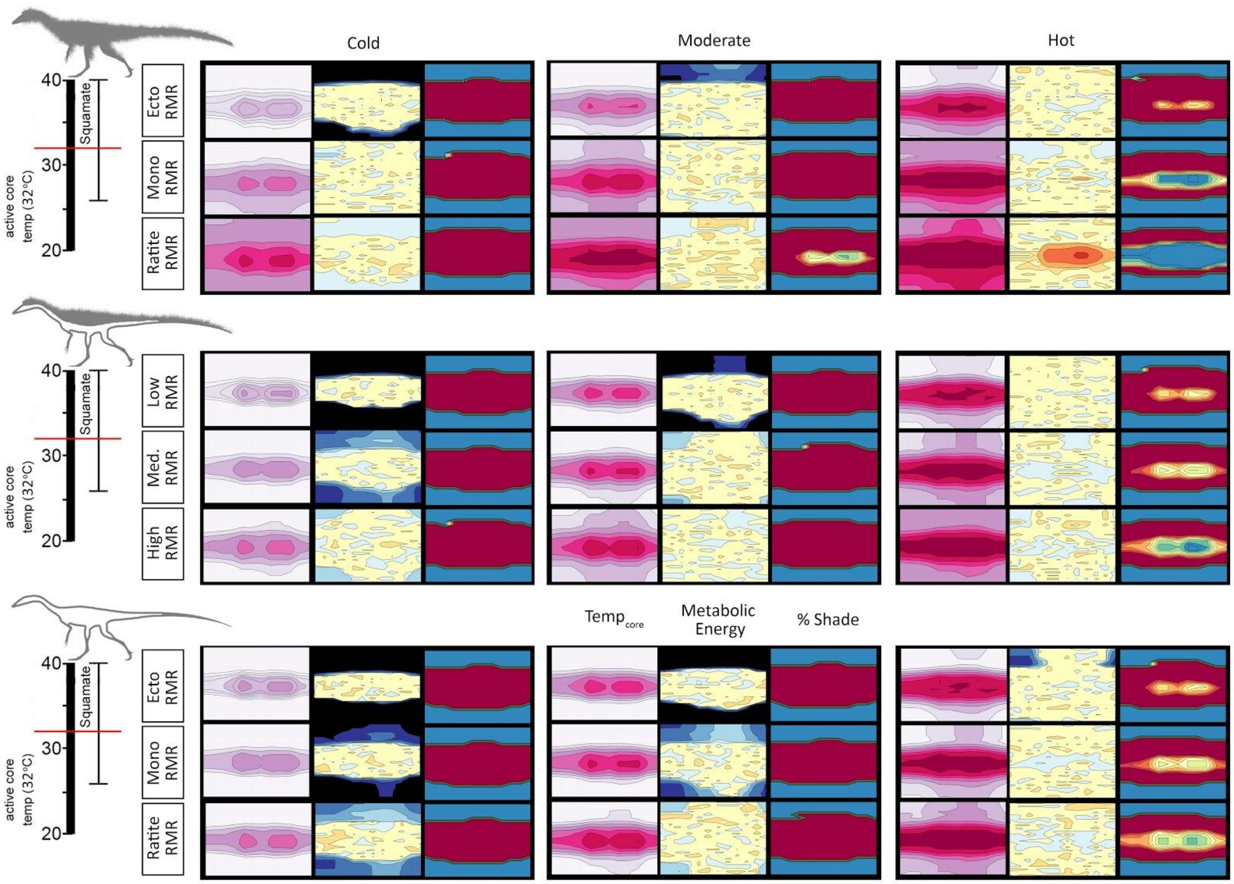

Figure 12. Heatmap of 32°C *target* T_{core} w/ broad CTR; *Coelophysis* (all).
With a broad CTR *Coelophysis* was able to maintain a *target* T_{core} of 32°C with a high RMR, fully insulated in cold and moderate microclimates and with moderate to no insulation in the hot microclimates. Average T_{core} was: 33.2°C with high RMR and full insulation in the cold microclimate, 32.5°C with high RMR and top-only insulation in the moderate microclimate, and 34.7°C.

Table 1. Average annual T_{core} *Coelophysis* (all).

Broad CTR 32 degree Target

	RMR	Microclimate		
		Cold	Moderate	Hot
Furry	Squa	27.6	29.3	32.9
	Mono	30.3	33.2	35.8
	Ratite	33.2	35.6	37.8
FurryTop	Squa	27.1	28.9	32.4
	Mono	27.9	30.1	34.4
	Ratite	28.9	32.5	35.6
Naked	Squa	27.3	29.0	32.0
	Mono	27.9	29.7	33.4
	Ratite	28.5	30.8	34.7

Broad CTR 38 degree Target

	RMR	Microclimate		
		Cold	Moderate	Hot
Furry	Squa	27.5	29.5	33.2
	Mono	31.0	34.3	37.5
	Ratite	36.7	38.5	39.0
FurryTop	Squa	27.1	28.9	32.5
	Mono	28.0	30.4	34.8
	Ratite	29.6	33.2	37.0
Naked	Squa	27.4	29.1	32.0
	Mono	28.0	29.9	33.5
	Ratite	28.8	31.2	35.0

Moderate CTR 38 degree Target

	RMR	Microclimate		
		Cold	Moderate	Hot
Furry	Squa	32.0	32.6	34.3
	Mono	33.2	34.6	36.7
	Ratite	36.1	38.1	38.8
FurryTop	Squa	32.0	32.3	33.8
	Mono	32.0	32.8	34.5
	Ratite	32.3	33.7	36.0
Naked	Squa	32.0	32.5	34.1
	Mono	32.0	33.0	34.5
	Ratite	32.3	33.6	34.8

Narrow CTR 38 degree Target

	RMR	Microclimate		
		Cold	Moderate	Hot
Furry	Squa	36.0	36.2	37.2
	Mono	36.4	37.0	38.0
	Ratite	37.5	38.6	39.0
FurryTop	Squa	36.0	36.1	37.0
	Mono	36.0	36.3	37.3
	Ratite	36.1	36.7	37.7
Naked	Squa	36.0	36.1	37.1
	Mono	36.0	36.3	37.3
	Ratite	36.0	36.7	37.5

References:

1. Smith NP, Barclay CJ, Loiselle DS. The efficiency of muscle contraction. Prog Biophys Mol Bio. 2005; 88(1): 1-58.
2. Farmer CG, Sanders, K. Unidirectional airflow in the lungs of alligators. Science. 2010; 327: 338-340
3. Benson RBJ, Butler RJ, Carrano MT, O'Connor PM. Air-filled postcranial bones in theropod dinosaurs: physiological implications and the 'reptile'–bird transition. Biol Rev. 2012: 87: 168–193.
4. Schachner ER, Cieri RL, Butler JP, Farmer CG. Unidirectional pulmonary airflow patterns in the savannah monitor lizard. Nature. 2013; 506: 367-370.
5. Maina JN. Functional morphology of the avian respiratory system, the lung–air sac system: efficiency built on complexity. Ostrich. 2008; 79(2): 117–132
6. Barton WH, Houston BC. A comparison of digestive efficiency in birds of prey. Ibis. 1993; 135(4): 363-371
7. Pough FH. Lizard energetics and diet. Ecology. 1973; 54: 837–844.

8. Swart D, Mackie RI, Hayes JP. Influence of live mass, rate of passage and site of digestion on energy metabolism and fibre digestion in the ostrich (Struthio camelus var. domesticus). S African J of Animal Sci. 1993; 23(5): 119-126.
9. Lopez-Calleja MV, Bozinovic F. Energetics and nutritional ecology of small herbivorous birds. Revista Chilena de Historia Natural. 2000; 73: 411-420.
10. Franz R, Hummel J, Kienzle E, Kölle P, Gunga, H-C, Clauss M. Allometry of visceral organs in living amniotes and its implications for sauropod dinosaurs. Proc Royal Soc B. 2009; 276: 1731-1736.
11. Shipley LA. Grazers and Browsers: How Digestive Morphology Affects Diet Selection. Idaho Forest, Wildlife & Range Exp. Sta. Bull. 1999; 70: 20-27.
12. Schrader AM, Owen-Smith N, Ogutu JO. Blackwell Publishing Ltd How a mega-grazer copes with the dry season: food and nutrient intake rates by white rhinoceros in the wild. Func Ecol. 2006; 20: 376-384.
13. Sellwood B, Valdes P. Mesozoic climates: General circulation models and the rock record. Sediment Geol. 2006; 190: 269-287.
14. Box GEP, Hunter WG, Hunter JS. Statistics for Experimenters. 1st ed. New York:John Wiley & Sons, Inc;1978.
15. McMahon TA. Using body size to understand the structural design of animals: quadrupedal locomotion. J Applied Physiol. 1975; 39(4): 619-627.
16. McNab BK. An analysis of the factors that influence the level and scaling of mammalian BMR. Comp Biochem Phys A. 2008; 151(1): 5-28.
17. McNab BK. Ecological factors affect the level and scaling of avian BMR. Comp Biochem Phys A. 2009; 152(1): 22-45.
