# Supplementary material for: Modeling Dragons: Using linked mechanistic physiological and microclimate models to explore environmental, physiological, and morphological constraints on the early evolution of dinosaurs
Source: PLoS One. 2020 May 29;15(5):e0223872. doi: 10.1371/journal.pone.0223872 (PMC7259893; doi:10.1371/journal.pone.0223872)
Supplement: S1 Table — (PDF) [file pone.0223872.s005.pdf]

S1 Table. Target resting metabolic rates for classes of animals.

Taxa	Mass (kg)	Tcore (C)	[1]	[2]	[2]	[3]	[1]	[2]	[1]
Class (target RMR) (W)			Squa	Mono	Mars	Ratite	Mars	Euth	Euth
Coelophysis	21	38	3.339	12.345	20.575	22.161	23.973	26.561	33.262
Plateosaurus	850	38	70.143	179.933	299.888	301.071	366.626	387.129	533.757
Varanus komodoensis	6.7	38	1.348	5.399	8.998	9.904	10.329	11.615	14.12

Reference [#], Class (watts)	
[1], Squa (squamate)	((0.202 10"(0.038 Tc-1.771))+M "0.82)/3.6
[2], Mono (monotreme)	(0.033*((M*1000)"0.724))*0.28
[2], Mars (marsupial)	(0.055 (M 1000)"0.724) 0.28
[3], Ratite	(0.07157 ((M*1000)"0.705)) 0.28
[1], Mars (marsupial)	(2187*M " 0. 737)"'(4.185/3 600)
[2], Euth (eutherian)	(0.071 (M*1000)"0.724) 0.28
[1], Euth (eutherian)	(70*M " 0.75) (4.185/(24 *3.6))

References

1. McMahon TA. Using body size to understand the structural design of animals: quadrupedal locomotion. J Applied Physiol. 1975; 39(4): 619-627.
2. McNab BK. An analysis of the factors that influence the level and scaling of mammalian BMR. Comp Biochem Phys A. 2008; 151(1): 5-28.
3. McNab BK. Ecological factors affect the level and scaling of avian BMR. Comp Biochem Phys A. 2009; 152(1): 22-45.
