# Supplementary material for: Modeling Dragons: Using linked mechanistic physiological and microclimate models to explore environmental, physiological, and morphological constraints on the early evolution of dinosaurs
Source: PLoS One. 2020 May 29;15(5):e0223872. doi: 10.1371/journal.pone.0223872 (PMC7259893; doi:10.1371/journal.pone.0223872)
Supplement: Attachment — Submitted filename: Review - Lovelace et al.pdf [file pone.0223872.s007.pdf]

Review: ***'Modeling Dragons: Using linked mechanistic physiological and microclimate models to explore environmental, physiological, and morphological constraints on the early evolution of dinosaurs'***

This paper uses the biophysical modelling software Niche Mapper to investigate the likely physiology and climatic tolerances of Late Triassic dinosaurs. These results are used to evaluate the hypothesis that large-bodied sauropodomorphs were precluded from lower-latitudes during the Late Triassic by high temperatures, finding that a metabolic profile permitting survivability at higher latitudes is inconsistent with the environment of the Chinle Formation. By contrast, the broad distribution of coelophysoids during the Late Triassic and Early Jurassic is used to suggest the variable development of feathery integument in these taxa, allowing them to survive a range of temperatures.

I do like what this paper is trying to achieve – establishing a quantitative, analytical protocol to test metabolic hypotheses in extinct taxa. If successful at this, Nice Mapper would undoubtedly be a very welcome addition to the palaeontologist's toolbox. Unfortunately, however, I have multiple concerns rising from conflicts between the results of this paper and the fossil record than mean I am presently unconvinced as to whether or not the models have accurately reconstructed the physiology of these dinosaurs. In particular, from the current result I do not think that Niche Mapper would resolve any non-insulated small archosaur as viable in the Triassic of Europe at all, which is at direct odds with the fossil record. This is fatal to the paper, given that one of its main roles is introducing this method to palaeontologists, but at current it is unclear whether this method is too imprecise to actually be useful. Further, although I do think the author's conclusions regarding thermal stratification of sauropodomorph communities and theropod integument are plausible, and indeed even reasonable, my concerns regarding the results means that it is presently unclear if they actually support these conclusions. In this case, the paper would not bring anything new to this discussion. Finally, I also have concerns regarding the polarity of discussion resulting in circular reasoning in the paper. Consequently, I must unfortunately advise rejection of the paper in its current form.

Still, I must reiterate that I do appreciate the goal of this paper, and applaud the effort that has been put into providing a broad suite of sensitivity analyses. I do have some suggestions for additional sensitivity analyses that would help convince me that the Niche Mapper experiments could be successful in explaining Late Triassic dinosaur distribution. These are explained below, along with my detailed comments on the paper. If these are addressed, I would be more than happy to review this paper again during a subsequent submission.

Detailed comments

In general

A key result of this study is that an uninsulated *Coelophysis* would suffer extreme cold stress in temperate latitudes, obligating insulating integument. Comparison of results with *Plateosaurus* and *Varanus* suggest these differences are primarily the result of size. From the results presented I am concerned that Nice Mapper would resolve any small, uninsulated archosaur/reptile more broadly as unviable in high-latitude Triassic environments, which would be inconsistent with the fossil record. For instance, crurotarsans of broadly similar ecology and body shape (e.g. *Ornithosuchus*) or subequal size (and in some cases much smaller – e.g. *Terrestisuchus*) to *Coelophysis* are known from temperate Triassic communities, despite their phylogenetic position making insulating integument highly unlikely. I acknowledge that these taxa were still anatomically different from *Coelophysis*… but they were more similar to it than *Varanus*, which is nonetheless resolved to exhibit similar model performance to *Coelophysis*, under common metabolic inputs. I appreciate that Niche Mapper is well validated from extant taxa, but we are dealing with a very different world and fauna here. This problem is compounded by ontogeny. Whereas adult *Plateosaurus* exhibits broad thermal tolerance due to its size, it had to spend years (~12; Sander & Klein, 2005) as a growing juvenile first. Hence, allometric effects, rapid growth and integument nonwithstanding, it must have been a viable organism for at least part of the year at *Coelophysis*-grade body sizes. It has been widely suggested that more deeply-nested sauropodomorphs exhibited dramatic shifts in metabolic rate through ontogeny: this could be part of an answer here but also highlights the massive uncertainties in this kind of study.

TL; DR: For me to be convinced that these analyses are representative, I would need some kind of sensitivity analysis to prove that a small reptile could work in a high-latitude Triassic fauna. This isn't arbitrary scepticism: there are many uncertainties in the inputs for these models, and so myriad sensitivity analyses are necessary to ensure that error bars are tight enough for them to be useful.

There is also bit of a disconnect between the polarity of the analyses performed and that described in the abstract, introduction and discussion. The experiments here ultimately use the distribution of *Plateosaurus* and coelophysoids, assuming (reasonably) that it was driven by temperature, to test between hypothesised metabolic regimes and integuments of these animals. This makes sense, as it is using observed data to inform more speculative traits. However, the abstract, introduction and discussion instead imply that metabolic data was used to test the importance of thermal stress versus other effects in structuring Triassic dinosaur distribution, when alternative hypotheses are not really evaluated. For example, Whiteside *et al*. (2015) suggested that metabolic requirements, rather than thermal stress, drove these patterns. The results here finding that *Plateosaurus* could easily make its metabolic requirements do refute this scenario, but this point does not receive adequate discussion at the moment given its importance. Other ecological/biological drivers are very hard to test, but they do need to be discussed. I know this comment on how the experiments are framed may sound like splitting hairs, but it is important to avoid circularity. Further, the discussion states that thermal stress was the "primary driver" on sauropodomorph distribution in the Late Triassic, but never makes the caveat that there are other potential drivers that are not tested here, or even readily testable at all.

Other comments:

Page 4: "For instance, it has been noted that there is an absence of large (>~1000 kg) prosauropod dinosaurs in the well-studied tropical to subtropical latitudes during the Late Triassic (e.g., the Chinle Formation of southwestern U.S.), while smaller (<~100 kg) theropod dinosaurs and their closest relatives are quite common." – This is true, and is the whole point of the paper. However, something that is not noted throughout is that small-bodied (~10kg) sauropodomorphs (*Thecodontosaurus*, *Pantydraco*) are known from higher latitude European faunas during the Late Triassic, but are also absent from the Chinle Formation. This raises two issues. First: I doubt that Niche Mapper would resolve these taxa as viable in the formations in which they are known to have occurred (see above). Second: what was precluding them from the Chinle Formation? Whereas the paper does make an

internally logical case for thermal stress excluding large sauropodomorphs from the Chinle Formation, the occurrence of these smaller-bodied taxa is still relevant as it raises the possibility of alternative forces limiting the distribution of sauropodomorphs more generally (but see comments below on trackways). At the very least, it provides a cautionary note on the suggestion in the main text that thermal stress was the "primary driver" of sauropodomorph distribution in the Late Triassic.

Page 21: "*Coelophysis* has long been considered a predatory theropod" – I think you can be bolder than this, given the known gut contents.

Page 24 (and supplementary information): "The parameters outlined above had relatively small effects on metabolic needs of the modeled organisms" – I applaud the range of sensitivity analyses performed, and do not have an issue with each being resolved to have a negligible overall effect. However, an estimate of the cumulative error/image of total error bars from these uncertainties would be helpful (in the supplemental information?) to help the reader gauge accuracy.

Page 45: "The daily temperature profile [for insulated *Coelophysis*] for the month of May with a squamate-like RMR and CTR is strikingly similar to that seen for a small adult komodo dragon (e.g., Fig. 11)." – This result seems to suggest that body shape has little overall effect on temperature profile. Is this reasonable? Or does it instead source from uncertainties in reconstructing original shape in the dinosaurs?

Pages 49: "The trackmakers would have been similar in size to the Early Jurassic skeletons of *Seitaad* (Sertich and Loewen 2010) and *Sarahsaurus* (Rowe and others 2011)." – It would be worth explicitly stating here that *Seitaad* and *Sarahsaurus* are both comparatively small (~100kg). Indeed, noting a size-dependent pattern is still notable in the Early Jurassic, with north American taxa (*Anchisaurus*, *Sarahsaurus*, *Seitaad*) being much smaller than the largest representatives from higher latitudes (e.g. Elliot and Lufeng Formation taxa) would only bolster your argument (although the obviously uneven sampling of these communities is a problem). Looking at your current results, I wonder if a 100kg *Plateosaurus* would be ok in a low-latitude setting anyway, obviating anything problematic about

these trackways even before elevation is considered – modelling this may hence be worthwhile. Indeed, this would actually help your argument, as it would prevent the otherwise paradoxical occurrence of small-bodied sauropodomorphs in higher latitude faunas alone from suggesting other causal mechanisms structuring 'prosauropod' occurrence.

Page 50: "Large size alone is not a limiting factor for the Late Triassic Chinle paleoecosystem." – This is true. You could, however, go a bit further by noting that, even then, the largest dicynodont taxon, *Lisowicia*, is known from European deposits. Shuvosaurids, which exhibit many convergences with dinsoaurs, also show a similar pattern, with small-bodied taxa from the Chinle Formation (*Effigia*, *Shuvosaurus*) but larger forms known from higher latitudes in South America (*Sillosuchus*). Although overall numbers of data points are quite low, discussion of these and other examples suggesting a phylogenetically broad occurrence of Bergmann's Rule in the Late Triassic would only help to bolster your arguments.

Page 52: "and similarly sized *C. rhodesiensis* is well known from the Elliot Formation (Zimbabwe) which was deposited in a temperate southern hemisphere paleolatitude… inhabited, varying the amount and location of insulation covering its body solves this apparent paradox." – Alternatively, was the Elliot Formation simply more moderate in temperature than the Lowenstein Formation? I know they were of comparable latitude, but palaeoclimatic data is not discussed. Another way of looking at this, while remaining in the same explicitly comparable Late Triassic time bin, would be to see how the *Coelophysis* model fares when scaled-up to a *Liliensternus*-sized individual? The integumentary argument already presented in the paper is reasonable, but surely the whole point of this method is that it allows us to exhaustively test these scenarios?

Page 52: "It is likely that that these structures were lost as sauropodomorphs increased in size - increased mass alone can expand tolerance of cooler temperatures and stabilize internal temperature variation, but not without its own energetic costs." – What about *Thecodontosaurus*? It is a small bodied (~10kg) sauropodomorph known from 35N Late Triassic sites. Presumably, the authors hold

that it would retain insulating integument as it precedes phyletic size increase within Sauropodomorpha (but see *Bagualosaurus*) but this does not receive any discussion, and would further highlight the numbers of assumptions still necessary to support their hypothesis. See also my comments above about juvenile *Plateosaurus*.
