# Supplementary material for: Modeling Dragons: Using linked mechanistic physiological and microclimate models to explore environmental, physiological, and morphological constraints on the early evolution of dinosaurs
Source: PLoS One. 2020 May 29;15(5):e0223872. doi: 10.1371/journal.pone.0223872 (PMC7259893; doi:10.1371/journal.pone.0223872)
Supplement: Attachment — Submitted filename: PONE-D-19-26881R1_reviewers_comments.pdf [file pone.0223872.s008.pdf]

Submission PONE-D-19-26881R1: Reviewer's response to authors' replies

In my previous review of this manuscript, I essentially only had one problem with it: I was concerned that the results within were inconsistent with allowing small-bodied archosaurs to have existed at high latitudes during the Triassic, which is at clear odds with the fossil record. I hence requested an additional analysis to prove to me that this was not the case, which would have allowed me to throw my full support behind the results.

In this resubmission, the authors have not included this analysis, as they argue that modelling additional taxa is beyond the scope of this study. Although I still think this would have been an effective way to increase confidence in the results and conclusions, I actually do take their point on this. Further, I reiterate that I appreciate that NicheMapper is well validated on extant taxa (but would add that the authors should not be surprised by additional scepticism when a method is extrapolated far beyond its test dataset). I also think that the rewriting of the paper has helped considerably to address some of my previous concerns regarding the polarity of the discussion.

Consequently, I am satisfied to recommend this paper for publication without this additional analysis. In its place, though, I really do think that the Discussion needs to contain an explicit defence of how animals broadly similar to *Coelophysis* and/or small prosauropods would have survived at higher latitudes. I doubt that I would be the only reader to draw these questions, and so it would only strengthen the paper to tackle them head-on. The good news is that the authors have already done so forcefully in their responses to me, citing a range of behavioural strategies, with some fossil evidence, as well as NicheMapper studies on extant taxa – incorporating this into the Discussion would only help the manuscript. I think that explicitly (if briefly) detailing the alternatives and corollaries here – that these compensatory mechanisms were widespread and/or integument may have been broadly distributed in Archosauria (albeit with the caveat that further testing is required in both of these instances) would really help give the reader what they need to evaluate the conclusions.

In summary, although I am disappointed to see some of the analyses I requested were not performed, I am prepared to admit that they are beyond the immediate scope of this contribution. Hence, I am satisfied with the paper pending expansion of the Discussion, including content from some of your responses to me, as outlined above. Were this to be completed, I would not feel that I would need to see this manuscript again, although would be happy to do so if the Editor deemed it necessary.

Responses to authors' replies

"Authors: However, we would point out that this method is not 'imprecise' as Reviewer #2 states. As a mechanistic model the precision is high and reproducible with the same input parameters, but lacking any significant means for absolute testing we cannot at this time measure the accuracy outside of bounding it by other means such as paleobiogeographic distribution, a phylogenetic framework, osteohistology and isotopic thermometry (which we do).

The values we use for climate have a broad range (not super precise, but the proxies are well vetted for accurately representing climate regimes). This is, in part, why we use three climate regimes (the state of our understanding of ancient climates in deep time is not highly precise, but given the error associated with climate proxies it is reasonably accurate). This paper is meant to show that a reasonably bound set of parameters can be used to test physiological (and behavioral) traits that provide a solution to the model, i.e., under currently known distributions and paleoclimates of the studied organisms (to the best of our knowledge at this time). Furthermore, the model can generate hypotheses that are predictive and falsifiable which makes it a valuable tool for deep time studies. At this time we cannot likely determine with high accuracy the exact physiology of extinct organisms, but we can quantitatively constrain their probable physiology based on known size, shape, phylogenetic position, geographic distributions, and paleoclimate. Other proxy data such as clumped isotope analyses and histology help further constrain the model and improve accuracy (see Padian and de Ricqlès, 2020). As new data arise, these may be incorporated to improve modeled results.

Another way to look at this is our Niche Mapper models in deep time produce a hypothesis, much like a phylogenetic analysis produces many equally most parsimonious hypotheses. We present all of the solutions from the model, and our interpretations of the most parsimonious solutions. The addition of new paleogeographic, isotopic, physiological or paleoclimatic data (additional OTU's or character/character states in the case of the phylogenetic analyses analogy) will likely lead to different results supporting or rejecting previous hypotheses, and new hypotheses will be generated until hopefully at some future point a consensus is reached. That is the power

of this model, and the point of this paper - the demonstration that combined mechanistic models of microclimate and physiology can be used in deep time to build, support, or reject hypotheses (emphasis added).”

I did not mean to imply that these results were not reproducible – that is clearly not the case. I also reiterate that I applaud the vision of this study, and I do understand that it is intended to generate hypotheses. My position was that, as the broader significance of this paper is presenting and advocating a novel method to the field, it is contingent upon that paper to demonstrate that the method works, assuage all reasonable doubts and provides novel perspectives relative to currently used methods. Hence, while we can all agree that the method results are precise, I was inquiring as to whether the inputs were so broad that the conclusions don’t really help us to narrow anything down further than what we already knew.

However, following the re-writing of the paper, which tweaks their question and emphasises the hypothetical nature of the results, I accept that my previous position is now too exacting. It remains true that NicheMapper is well validated on extant taxa, and many sensitivity analyses are performed, so I do now think that the paper satisfactorily addresses its (refined) goals, and so it does represent an adequate case-study.

Detailed comments

Authors: “We can appreciate the concern that Reviewer #2 has regarding the possible success or failure to model other small archosaurs (or reptiles) at high latitudes under the same climate conditions as outlined by our modeled Triassic environments. We agree that IF our model could not resolve smaller reptiles that are known from the high latitude fossil record that would call into question the reliability of the model. However, as Reviewer #2 has noted (below) they are aware that Niche Mapper is well vetted for a very broad range of extant organisms, including small reptiles. Moreover, Niche Mapper readily incorporates burrowing, nesting, climbing trees, and hibernation as a behavioral means of thermoregulation, torpor (or aestivation), basking, etc.”

Reviewer: As previously noted, I do acknowledge that NicheMapper has been successfully applied to small reptiles. It is for this reason that I thought it would be relatively trivial to fit these data to a Lowenstein climate to put any concerns that anybody could have to rest*.

As this is apparently not the case, however, I think it would at least be appropriate to explicitly discuss this point in the Discussion – by stating that we know smaller-bodied reptiles existed in these

environments, but would have survived via these behaviours, as seen in extant taxa (citing a NicheMapper study of a high-latitude reptile would be useful here).

*Again, I do believe that NicheMapper is useful, and I think your conclusions are reasonable. It would just be useful to address this possible source of discrepancy.

"Several of these behavioral traits were not modeled for Coelophysis *or* Plateosaurus *because it is unlikely that either of these dinosaurs were burrowing or hibernating (Sanders, et al., 2004; Klein and Sanders, 2007; Griffin and Nesbitt, 2016). That is not to say that all dinosaurs did not burrow, several high latitude dinosaurs are in fact specialized for it (Varicchio et al., 2007; Martin, 2009). It is likely that small reptiles/other archosaurs living alongside Plateosaurus would be viable if allowed to burrow without invoking dermal insulation such as hair or feathers. Several clades of high latitude Triassic vertebrates are also known to be burrowers..."*

Reviewer: Fair enough that you have not modelled these behaviours in these dinosaurs. Again, though, it would be worth citing the broad distribution of burrowing behaviour in Triassic taxa in the discussion to help bolster your case.

Authors: "Agreed - Ornithosuchus *and* Terrestisuchus *are more similar in ecology/body shape than Varanus but there is actualistic data for* a *which is why we included it as a cursory test for the modeling of a large-bodied squamate (to ensure* Coelophysis *and* Plateosaurus *modeled as squamates would be reasonably accurate)."*

Reviewer: I did not mean to critique your inclusion of *Varanus* data – I just meant that its similar performance to *Coelophysis* forces to me to conclude that *Ornithosuchus* etc. would perform in a similar way in these models.

"Again, the suggestion to model crocodylomorphs is a great idea and well worth pursuing in a followup paper(s). It would be quite telling to quantify the benefit a shallow, moderate, and deep burrow might provide a rather gracile Terrestrisuchus *compared to an individual without the benefit of a burrow. In fact, it would be a great future project to model Alligator geographic distribution with and without the ability to burrow/den. It is*

certainly well established that Alligator (and Varanus *) use dening/burrowing to survive wildfires, and extreme weather such as significant cold spells and hurricanes (I would speculate at this time that without the ability to den it would not likely survive nearly as far north as it currently does), as well as aqueous diving behaviors to thermoregulate in extreme heat; these fossorial habits may well be a holdover from their crocodylomorph ancestors, e.g: "The occurrence of almost complete articulated skeletons in nearby correlated stratigraphic levels suggests they could dig large, deep burrows in soft substrates that allowed, like living alligators, thermoregulation. These excavated holes provide a source of water during dry periods. This ethological aspect probably allowed them to live in more terrestrial and arid environments." - I.S. Carvalho et al., 2005,* Baurusuchus salgadoensis *, a New crocodylomorpha from the Bauru Basin (Cretaceous), Brazil. Gondwana Research; 8(1): 11-30.*
It should be noted too, that while Terrestrisuchus *and its relatives may not have had epidermal insulation (currently phylogenetically constrained to Ornithodira), either croc-line archosaurs or archosaurs in general may have had elevated resting metabolic rates based on respiratory anatomy (Brocklehurst and others, 2020), upright locomotion (Seymour and others, 2004) and inferred growth rates (Legendre and others, 2016). Investigating the impact of those metabolic inferences on non-dinosaurian archosaurs is an exciting avenue of potential research, for ourselves and others who adopt mechanistic modeling techniques."*

Reviewer: I suppose you are right that modelling these taxa is beyond the immediate scope of this paper. I also think that these suggestions for how these taxa could have coped with such conditions – behavioural or integumentary – are plausible, and so should be mentioned. The content in your reply here is valuable, so why not use it in the discussion itself? I do not think I would be the only reader who would be left wondering about the repercussions for other small-bodied taxa and, even if modelling them is beyond the scope of this study, you could at least briefly address this by commenting that even though similar taxa did occur in those localities, they may have used these different strategies.

Authors: "● We added: " Likewise, phylogenetically disparate but spatiotemporally contemporaneous species should also be incorporated in future studies to further test ecological hypotheses. " at the end of the discussion to state

this. Additional supporting comments are made in the discussion that caveat what we know, and what we are lacking or need to improve upon."

Reviewer: This extra discussion point is certainly appreciated on my part. However, I do think that it needs to be expanded upon. I will take your point that modelling these other taxa is beyond the immediate scope of the paper, but I do think they at least need to be addressed, "e.g. a potential discrepancy is the presence of similarly-sized pseudosuchians in these localities… small-bodied extant taxa use behaviours such as burrowing to survive such conditions. There is broad evidence of burrowing in Triassic taxa, and may be a common strategy in pseudosuchians. Alternatively, epidermal integument may be more broadly distributed in Archosauria than currently thought." I do not think I will be the only reader will be left conflicted by the presence of these taxa in areas too cold for uninsulated theropods, so just demonstrating that you have thought in depth about how they would have compensated goes a long way to strengthen your arguments. It also ensures that everything is explicitly presented to the reader so that they are best placed to evaluate the overall conclusions.

Authors: "Niche Mapper does not assume any particular physiology, anatomy, or environment, and its inputs can be made as different or similar to modern examples as the evidence allows. Beyond that it is simply calculating thermodynamic processes, which surely were not significantly different in the Triassic than they are today. On the contrary, Niche Mapper provides a tool for quantitatively testing how different taxa and environments could have been. For instance, RMR and CTR combinations outside of those observed in modern organisms can be quantitatively tested against paleobiogeography and paleoenvironmental datasets. Conversely, paleobiogeography and paleophysiology can be tested against paleoclimate data to derive quantitative estimates, and the impacts of parameters that we currently have no proxies for (i.e. cloud cover). While the climates, flora and fauna of the Triassic were different from today, there is little reason to think those differences extended to cellular function, or that local climates could have had conditions physically impossible in the modern world."

Reviewer: Again, I do not doubt this – NicheMapper's broad validation and reliance on fundamental physiological parameters are strengths. Still, extrapolating this far back into the fossil record is still an extraordinary application, so you should expect additional scepticism.

Authors "This point has been taken into consideration and we added 'Ontogenetic shifts in physiology (e.g., declining metabolic rates with age/size),

thermoregulatory behavior, or insulation (e.g., dermal or subdermal) should all be taken into account in future work. Clearly, hatchlings through sub adult members of a population must have survived to adulthood in our modeled climates, whether through physiological, environmental, or behavioral means. Likewise, phylogenetically disparate but spatiotemporally contemporaneous species should also be incorporated in future studies to further test ecological hypotheses' to the end of the discussion."

Reviewer Thank you for including this. Many paleobiological analyses based on adults seem to forget that juveniles also have to be functional, so I am glad to hear you have ongoing research in this area.

Authors: "This is addressed above - there is a broad body of research (see Additional Modeling References below) that support small reptiles in a variety of climates with high accuracy. The problem isn't that a small reptile (or juvenile dinosaur) wouldn't work in a high latitude model - they do - it's just that they are inherently different with different solutions to thermoregulation than an adult Coelophysis *or* Plateosaurus*."*

Reviewer: I still think it would have helped your case in the first instance by showing this as opposed to just telling it. While I now accept that such an analysis is beyond the immediate scope of this study, I nevertheless do think this point should be more explicitly discussed (see above).

Authors: "We appreciate this critique and have restructured the Abstract (significantly) and Introduction (minorly) to reflect this. The addition of " The paleobiogeographic distribution of Plateosaurus and Coelophysis along with associated paleoclimate proxies from known fossil localities provide boundary conditions to test hypotheses of environmental exclusion due to physiological limitations. " in the last paragraph aligns the polarity of the introduction for a clearer flow.
The discussion is reworked as well, incorporating several of the suggestions and critiques outlined throughout the reviewers comments.
Please see the 'marked up' version of the MS for changes - mostly they were reformatting the order of statements to make a more consistent and logical flow as suggested."

Reviewer: Thanks for doing this. I think that this rearrangement (and the discussion of your hypotheses that includes what would falsify them) really helps the submission.

Authors: "We added: " Whiteside and others [28] argued for climate driven environmental and vegetative instability as a dominant factor for the exclusion of large dinosaurs from the Late Triassic tropics. Our results suggest heat stress alone should have been a significant barrier for Plateosaurus-sized dinosaurs. We suggest environmental temperatures limited large bodied prosauropods from greater appearance in tropical latitudes during the Late Triassic."

Reviewer: Again, thanks for doing this. I am similarly satisfied with your responses to my other comments.

Authors comments on the manuscript:

Comment 6: *"attributed to a larger sauropod dinosaur" -> "but what about precision of identification?"*

Reviewer: I think it is fine as-is.

I have nothing to add on any of the other comments.
